# Health System and Individual Barriers to Supporting Healthy Gestational Weight Gain and Nutrition: A Qualitative Study of the Experiences of Midwives and Obstetricians in Publicly Funded Antenatal Care in Tasmania, Australia

**DOI:** 10.3390/nu16091251

**Published:** 2024-04-23

**Authors:** Michelle L. Kilpatrick, Alison J. Venn, Kristine R. Barnden, Kristy Newett, Cheryce L. Harrison, Helen Skouteris, Andrew P. Hills, Briony Hill, Siew S. Lim, Kim A. Jose

**Affiliations:** 1 Menzies Institute for Medical Research, College of Health and Medicine, University of Tasmania, Hobart, TAS 7000, Australia; alison.venn@utas.edu.au (A.J.V.); kim.jose@utas.edu.au (K.A.J.); 2 Centre for Mental Health Service Innovation, Advocate House, Hobart, TAS 7000, Australia; 3 Royal Hobart Hospital, Hobart, TAS 7000, Australia; kristine.barnden@ths.tas.gov.au (K.R.B.);; 4 Monash Centre for Health Research and Implementation (MCHRI), Faculty of Medicine, Nursing and Health Sciences, Monash University, Melbourne, VIC 3168, Australia; cheryce.harrison@monash.edu; 5 Health and Social Care Unit, School of Public Health and Preventive Medicine, Monash University, Melbourne, VIC 3004, Australia; helen.skouteris@monash.edu (H.S.); briony.hill@monash.edu (B.H.); 6 Warwick Business School, University of Warwick, Coventry CV4 7AL, UK; 7 School of Health Sciences, College of Health and Medicine, University of Tasmania, Launceston, TAS 7248, Australia; andrew.hills@utas.edu.au; 8 Eastern Health Clinical School, Monash University, Melbourne, VIC 3128, Australia; siew.lim1@monash.edu

**Keywords:** pregnancy, nutrition/diet, physical activity, weight/obesity, behaviour change, antenatal care

## Abstract

Individual and health system barriers can impede clinicians from supporting weight-related behaviour change for pregnant women, particularly in publicly funded antenatal care accessed by women from diverse socioeconomic backgrounds. The aim was to understand clinicians’ experiences of supporting healthy gestational weight gain for pregnant women in a publicly funded antenatal setting. The work was undertaken to guide the implementation of systems changes, resource development, and workforce capacity building related to nutrition, physical activity, and gestational weight gain in the service. The qualitative descriptive study used purposive sampling and semi-structured interviews conducted between October 2019 and February 2020. Nine midwives and five obstetricians from a publicly funded hospital antenatal service in Tasmania, Australia participated. Interview transcripts were analysed using inductive thematic analysis. The three dominant themes were prioritising immediate needs, continuity of care support weight-related conversations, and limited service capacity for weight- and nutrition-related support. The subthemes were different practices for women according to weight and the need for appropriately tailored resources. Improving access to continuity of care and clinician training, and providing resources that appropriately consider women’s socioeconomic circumstances and health literacy would enhance the ability and opportunities for clinicians to better support all women.

## 1. Introduction

Pregnancy is a critical life stage when women may be more motivated to adopt healthier lifestyle habits with increased receptivity to health advice and behaviour change support from their antenatal care providers [1,2]. Weight-related behaviours such as diet and physical activity and healthy gestational weight gain (GWG) can influence short- and long-term physical and mental health outcomes for both mother and child [3,4,5,6]. Pregnant women who adopt healthier behaviours, for example, healthy dietary patterns or physical activity, may reduce their risk of excessive GWG [7], gestational diabetes [8,9], preeclampsia [10], and delivery complications [11]. Importantly, healthy nutrition and physical activity behaviours adopted by women during pregnancy may continue postnatally, improving the health of mothers as well as their offspring [12,13].

Midwives and obstetricians play an important role in supporting pregnant women to adopt healthier behaviours [14,15]. Australian and international clinical practice guidelines recommend that all women are supported during antenatal care to optimise their nutrition, physical activity, and achieve healthy levels of GWG [16,17,18], yet gaps often exist between the guidelines and clinical practice. GWG screening and counselling to support healthy GWG are not provided consistently [19,20,21,22,23,24], with commonly identified barriers including lack of clinician training and confidence, time pressures within appointments, a lack of continuity of care (where care is provided by a clinician or small team of clinicians known to a woman throughout her pregnancy, birth, and postnatal care), and concerns about damaging rapport [19,25,26]. It is recommended that this support be provided with consideration to the pervasive health system and care provision expressions of weight stigma which have been linked to a decreased quality of reproductive healthcare, and poorer health behaviours and pregnancy outcomes [27].

Women living with social and economic adversity can experience additional challenges to making positive changes to their health behaviours [28,29,30,31,32,33]. The World Health Organisation recommends that antenatal clinicians ensure that they understand a woman’s circumstances and provide support without stigma when discussing diet, physical activity, and GWG [4]. However, less is known about the provision of weight gain support during routine antenatal care appointments in the face of potentially competing health risks, and psychosocial and economic challenges. This research was undertaken in Tasmania, an Australian state that has greater levels of socioeconomic disadvantage than the Australian average. Compared to the rest of Australia overall, Tasmanians have lower rates of educational attainment, the highest levels of people living on government income support, the highest rates of teenage mothers, and the highest proportion of Australian women who begin their pregnancy living with obesity [34,35,36]. In 2021, 28% of Tasmanian mothers were living with obesity, higher than the Australian average of 23% [36]. In addition, nearly two thirds of Tasmanians have been estimated to lack the health literacy skills they need to manage their health and wellbeing [37]. This article presents a component of a broader examination of how antenatal clinicians at the largest publicly funded hospital in Tasmania provided nutrition, physical activity, and healthy GWG support to pregnant women. The aim of this component of the study was to examine clinician perceptions of personal and health system barriers to supporting weight-related behaviour change (nutrition and physical activity) and healthy GWG across all pathways of care, including pregnant women living with more complex health and psychosocial issues, and economic disadvantage. This formative work was conducted to inform system changes to the antenatal service, resource development, and workforce capacity building. Therefore, the article provides a pragmatic example of using research and evidence to guide intervention planning and implementation for health service change.

## 2. Materials and Methods

### 2.1. Design

The study used a qualitative descriptive design.

### 2.2. Setting

The research was conducted at the antenatal clinics of a public hospital in southern Tasmania servicing approximately 2000 births per year. The hospital is a state-wide tertiary referral centre for high-risk pregnancy planning and management, and supports community-based midwifery-led clinics that provide the continuity of care for women classified to be of lower clinical or psychosocial risk. All other clinics are held adjacent to the hospital where women are seen by obstetricians and midwives without the continuity of care (also referred to as non-continuity models of care) at the time the study was conducted. These clinics included a general medical clinic (a team approach between obstetric doctors and midwives); high risk (multidisciplinary care for women with medical or obstetric risks including a BMI over 50 and multiple pregnancy); complex care (multidisciplinary care for women with complex mental health, substance use, and psychosocial issues); endocrine (multidisciplinary care for women with diabetes or other endocrine disorders); and Young Mum (midwifery-led clinic for women under 19 years). There are no socioeconomic-related data collected by the hospital or elsewhere for pregnant women receiving their antenatal care through the hospital.

### 2.3. Participants

At the time the research was conducted, there were 14 obstetric staff (almost all female) and 27 female midwives working in antenatal care. We invited all midwives and obstetricians in the service to express interest in being part of this qualitative study, with no exclusion criteria. From those who expressed interest, we invited a representative cross-section of midwives and obstetricians to be interviewed who worked across all antenatal clinics [38]. Except for the midwives working in the community-based continuity of care clinics, hospital-based clinicians worked across multiple clinics, so they drew on their experiences working across a range of clinics.

### 2.4. Data Collection

Semi-structured interviews were conducted between October 2019 and February 2020 as part of a research project, with interviews conducted until no new ideas were discussed (data saturation) [39]. The interviews took between 20 and 30 min. The interview schedule [see Supplementary Table S1] was adapted from a UK-based study exploring obstetricians’, midwives’, and general practitioners’ approaches to weight management in pregnant women, with permission from the authors [40]. Changes to the schedule were made in consultation with clinicians on our study investigator team to ensure it was relevant to the local context. Face-to-face interviews were conducted in offices at the hospital and community antenatal clinics by the female lead author (MK) who is an epidemiologist with qualitative research experience. The interviews were audio-recorded, and they were conducted with clinicians until no new ideas were discussed, in line with recommendations on non-probabilistic samples and thematic exhaustion [41].

Ethics approval was granted by the University of Tasmania’s Health and Medical Human Research Ethics Committee (reference number H0017949), and all participants provided written consent.

### 2.5. Analysis

Interviews were transcribed, checked, de-identified, and imported into qualitative data management software NVivo 1.0 (QSR International, Doncaster, Victoria, Australia) for analysis. An inductive coding approach was used by MK on all transcripts to identify and generate codes. The codes were then reviewed and grouped into potential themes. Comparative independent coding and discussion of the themes was completed for one midwife and one obstetrician interview transcript by an experienced qualitative researcher (KJ) and MK. Themes were reviewed and refined by KJ and MK following further discussion, until there was agreement. Where there were distinct variations within a theme, the subthemes were identified to provide a more nuanced understanding of the overarching theme. The coding and analytic decisions were recorded in the project log. The interviews captured data on a broad range of factors related to weight, nutrition, and physical activity in antenatal appointments, and not all data are presented in this article.

### 2.6. Trustworthiness

The researchers endeavoured to ensure the study’s trustworthiness in several ways [42]. For dependability and confirmability, a detailed audit trail was kept. For credibility, a process of sensemaking and member checking was used [43], where the themes identified were considered for their importance and implications by clinician co-investigators and working group members. The themes were also presented to clinicians who had not participated in the study for confirmation that the analyses and themes identified were appropriate and relevant. This process in conjunction with prolonged engagement in the setting shaped the interpretation of the results and recommendations for action.

## 3. Results

Nine female midwives (three working in continuity of care models and six working across non-continuity models) and five female obstetricians (all working in non-continuity of care models) were interviewed. This was approximately one third of the midwives and obstetricians working in antenatal care at the time. The average length of experience for the midwives was 15 years (ranging from two to 38 years) and obstetric staff was 10 years (ranging from five to 18 years).

Data were collected for the full semi-structured interview schedule, and a component of the results that focused on the experiences of behaviour change support across the various clinics and needs of women are reported here. The results of this study draw together the combined midwife and obstetrician experiences due to the similarity of themes across clinician types, so the term clinician is used herein to refer to midwives and obstetricians collectively. The thematic content analysis identified three over-arching themes, with two subthemes.

**Theme** **1.** *Prioritising immediate concerns.*

Clinicians felt that health promotion in general as part of antenatal care was important for all women. Some expressed that women with multiple behavioural risk factors and complex life circumstances had the potential to benefit most from health promotion during their antenatal care.

*“I think [health promotion] is almost even more important because the complex care patients are more vulnerable and less knowledgeable I think, or less focussed on those aspects of their lives.” [Obstetrician 5, non-continuity of care (non-CoC)]*

However, clinicians reported that they were less likely to offer support about weight-related behaviour change when seeing women at higher risk or complex care clinics. Support around maintaining a healthy diet, being physically active, or achieving healthy weight gain was typically prioritised less or not at all by clinicians for women with complex health or psychosocial issues, resulting in inequality in terms of the opportunity for these women to receive diet and physical activity support. Clinicians talked of using a harm minimisation approach to focusing on behaviours with higher-perceived immediate risk to women and their babies than weight- or nutrition-related topics.

*“I saw a woman today, she’d just finished marijuana use, well done, done a great job. Still smoking 10 cigarettes a day, that’s okay, we’ve still cut down, we’ve stopped marijuana. Drinking two litres of Coke and coffee– she’s not obese, she’d be overweight but she wouldn’t be obese, not a healthy girl by any standards. How many things–how do I go about without it becoming a barrier, how do I go about talking about it all—so I congratulate her on the marijuana, that’s fantastic; let’s talk about the smoking today. Touch a little bit gently on the caffeine. Should I talk about the sugar? No. Not today.” [Midwife 8, non-CoC]*

Less time might be allocated to weight-related behaviours if a clinician perceived that a woman had competing issues that took precedence.

*“Yes, well, it tends to be a harm minimisation rather than achieve very much in those women…So, we do spend some time trying to encourage them to eat healthy, but we don’t spend a lot of time on it because there’s no point, if they’re distracted by dealing with their abusive partner and difficult children and getting drugs and trying to get more fresh vegetables is very low on the scheme of things. So, although we encourage them to eat more healthy food, it’s a relatively low priority in the scheme of things.” [Obstetrician 1, non-CoC]*

Clinicians might not address weight or weight-related behaviours at all if they believed that a woman might be overwhelmed by psychosocial or substance use issues, while several felt that supporting healthy weight gain and nutrition was not appropriate for women with higher-risk pregnancies.

*“So, there’s certain women that actually, it’s the last thing on their mind…they’ve got so much social stuff going on that actually for them to get to this appointment was so hard…Sometimes it’s really tricky to actually get in there and get past the typical illicit drugs, smoking, alcohol. Talking about weight or even healthier food choices, you can tell sometimes it’s a no-go. So, yes, I can be choosy. I won’t bring up something with someone in that consult if I can already tell it’s the least of their worries.” [Midwife 7, non-CoC]*

*“I think the women that would be better perhaps targeted [for healthy weight gain support] are ones that are having healthy pregnancies, because…like I saw a woman on Wednesday who has just been diagnosed with gestational diabetes but she’s got a baby with a major heart problem and so she just couldn’t even contemplate coming and thinking about the diabetes complication or diet for her pregnancy because she is just not in a good headspace.” [Obstetrician 2, non-CoC]*

**Subtheme** **1.** *Different practices for women according to weight.*

It was more commonly reported for clinicians to monitor weight gain and provide GWG support if a woman was living with overweight or obesity, indicating that a higher priority might be given to these practices if a woman presented with a higher body mass index (BMI). For some, this reflected a belief that monitoring weight was more important to address for these women.

*“I think it’s the ones that start off with the high BMIs that we need to be keeping a closer eye on.” [Midwife 5, non-CoC]*

*“[We weigh those] Who have got higher—bigger BMIs, yeah. Because the other women we don’t weigh. So, if they have a normal BMI, they don’t get weighed at all.” [Midwife 3, CoC]*

The weighing of women and nutrition support was more often reported when a woman was living with overweight or obesity, despite some clinicians talking of a higher number of women with insufficient nutrition and weight gain linked to women experiencing low economic status.

*“To be honest, I usually do that [talk about diet or activity] for ladies who’s overweight or obese or morbidly obese…because it’s important and it make a huge impact on their pregnancy and their delivery as well, as well as their life.” [Obstetrician 4, non-CoC]*

**Theme** **2.** *Continuity of care supports weight-related conversations.*

Overall, clinicians working with continuity-of-care models were more likely to engage in conversations around weight-related behaviours and healthy gestational weight gain than clinicians working with non-continuity models. The continuity of care was seen to provide an opportunity to build rapport and trust for all women.

*“They’re more likely to divulge things too if they’re comfortable. And it might take a couple of visits”. [Obstetrician 2, non-CoC]*

*“So, continuity of midwifery care is… a great way of building trust and helping to get across some of those health messages through antenatal care.” [Midwife 4, non-CoC]*

Clinicians working with continuity-of-care models acknowledged the benefits of being able to follow progress with all woman, with some noting that they used their established rapport with a woman to raise weight-related topics, reinforce health messages, and provide support multiple times throughout the course of antenatal care.

*“There is a few at the moment that I’m like, maybe you could do a bit more walking and things like that, me suggesting drinking water rather than drinking cordial and drinking Coke and those sorts of things. They’re going to keep coming and seeing me so…I’m a bit of a broken record when it comes to those things.” [Midwife 1, CoC]*

The monitoring of GWG was carried out more often with continuity-of-care models where clinicians reported basing their assessment of weight gain on the ability to visually judge change from appointment to appointment. A noticeable change might then prompt a woman being invited to be weighed.

*“The beauty of continuity is visual so you can tell and judge.” [Midwife 1, CoC]*

In contrast, clinicians working with non-continuity-of-care models were more likely to describe concerns about damaging rapport when discussing weight-related health behaviours with women. Several cited the risk of women disengaging from antenatal care because of different clinicians bringing up the same topics at multiple appointments without the benefit of continuous support.

*“Everyone talks the same thing, because that’s what we are supposed to do. So be it the midwife, be it the resident, be it the registrar, or a consultant, we try to touch those bases every time we see a patient. The patient, if you see from the other side of the table, they’re like why everyone be telling me that? I know that. I know that, please stop it, I’m aware.” [Obstetrician 3, non-CoC]*

Many working with non-continuity-of-care models described being unaware of what behaviour change advice or support had been previously given to a woman, as these details were not routinely recorded in patient records or were recorded with varying levels of detail. This made it difficult for clinicians to avoid the repetition of advice, and provide relevant and effective support at subsequent appointments.

*“It might not be documented at all. Or it could be “discussed diet and lifestyle,” “this woman exercises regularly, has a varied diet,” or, “discussed ways to modify diet to minimise weight gain, including limiting carbs,” or, “history of low iron last pregnancy; talked about high-iron foods this pregnancy.” [Midwife 6, non-CoC]*

Clinicians believed that women with more complex needs would benefit most from having the continuity of care to better engage with them about their health and weight-related behaviours, but noted the challenges these women faced by being allocated to complex care or high risk clinics which did not provide the continuity of care.

*“I feel for the complex care ladies because I think they’re the ones that need [continuity of care] the most in some ways because they don’t engage, they’ve got lots of social things going on and they sort of need someone on their side, if you know what I mean. And it’s much easier to get someone engaged in their health on a personal level rather than seeing whoever’s on [Midwife 8, non-CoC]*

Clinicians valued the continuity of care to better engage with women about their health and pregnancies. Those working with the high risk and complex care non-continuity models described trying to establish continuity in care where possible by opting to see women they recognise from previous appointments.

*“Where possible we try and get, you know, you’ve seen that person last time, can you see them again?” [Obstetrician 2, non-COC]*

*“You don’t have to go through the story every time…I will usually try and catch the eye of a few and if I know their name on the list I will try and grab them. [Midwife 8, non-CoC]*

**Theme** **3.** *Limited capacity for weight- and nutrition-related support.*

Overall, none of the clinicians interviewed recalled having undertaken pre-clinical or professional development training around nutrition in pregnancy, nor physical activity, weight, or behaviour change strategies.

*“…there’s just such minimal training about nutrition, which is a shame.” [Midwife 2, CoC]*

Clinicians expressed a need for more training, including evidence-based behaviour change techniques, nutrition, gestational weight management, and respectful ways to discuss weight with women.

*“I think the issue that I find tricky that I think we all need a little bit of help with is talking to women who are overweight about their weight” [Midwife 5, non-CoC]*

*“…maybe motivational interviewing techniques and goal setting, and a bit more about the current evidence around maternal weight management and weight gain.” [Midwife 4, non-CoC]*

While the majority felt confident advising on basic nutrition, clinicians were less confident with women with more complex dietary requirements or socioeconomic circumstances (i.e., food insecurity).

*“I know I can talk about eating more fruit and vegetables and less white stuff which is basically what I do when I talk to women about it. But when it comes down to the nitty gritty of what that actually means for this person in their situation, [we] don’t have the resources that allows us to do that.” [Obstetrician 1, non-CoC]*

As a priority, all clinicians wanted timely access to dietetic support, particularly for women with complex nutritional needs or circumstances that might affect the woman’s health or that of her child. Clinicians described having no viable referral pathways for women to access dietitians working within the hospital which would provide them with free support. While it was possible to make a referral, due to limited appointment availability, clinicians reported that appointments were not until late in pregnancy or more often the post-partum period. As such, clinicians typically did not refer women except in the most extreme cases and rarely to support healthy GWG.

*“At the moment, dietitian referrals don’t get acted on appropriately…There are no pathways for involving another service to help with lifestyle during a woman’s pregnancy [Midwife 7, non-CoC]*

*“Even people with severe nutrition issues struggle to get in to see a dietitian.” [Obstetrician 1, non-CoC]*

*“We’d love to have dieticians available…especially for women who either are really high or really low BMI.” [Midwife 6, non-CoC]*

Clinicians highlighted the importance of having dietitians as part of multidisciplinary antenatal teams, particularly for complex care and high risk clinics. Many described the advantages of having the integrated support available to women, as well as an accessible source of expert guidance for the midwives and obstetric staff.

*“With our high-risk clinic—so, these are women from all different sorts of categories of high-risk, and weight can be one of them—Where’s our dietitian? That’s something that I would vision to be part of the team. We so need that input for particular clinics.” [Midwife 7, non-CoC]*

Facilitating access to private dietitians with the cost covered by the antenatal service was seen as difficult, and no clinician reported having done so.

*“It’s been said that maybe for these high-risk women we can look at exploring referrals outside the public setting to private dietitians and that the hospital would somehow then fund. But logistically I don’t know how we would go about that.” [Obstetrician 2, non-CoC]*

**Subtheme** **3.** *Need for appropriately tailored resources and support.*

Clinicians described not having easy access to appropriate weight- and nutrition-related resources for women with different cultural backgrounds, low health literacy levels, and literacy levels.

*“You really do [have to make it simple] because I think the health literacy is very low here” [Obstetrician 5, non-CoC]*

*“I think we could do a lot better in some more accessible resources for women from not just low literacy but also languages other than English because just because they know the languages it doesn’t mean they read that language either so it’s something to work through. I think just visual things would work.” [Midwife 1, CoC]*

The importance of understanding the woman’s economic context when providing support was noted, including making affordable and practical recommendations.

*“I always try to encourage walking. I think that’s usually easy and important. And yeah, cheap.” [Midwife 9, non-CoC]*

*“We know that healthy options often aren’t cheap options, but it’s learning about how to put together a meal on budget, a healthy meal on budget. And then if your midwife says, “Oh, your iron level’s low,” you have to start on some iron tablets that cost $20 per bottle of iron. But that means the children will have to go without, and I’m not going to do that. So I won’t start the iron. And I can’t afford the red meat. So there’s inequity, yeah, that real health inequity. Those social determinants of health I think are often underestimated. And yet they’re so important.” [Midwife 6, non-CoC]*

## 4. Discussion

We aimed to explore clinician experiences of supporting diet and physical activity behaviour change and healthy gestational weight gain in publicly funded antenatal care, which includes providing care to women living with a range of health and psychological needs from diverse social, economic, and cultural backgrounds. The themes identified were clinicians prioritising nutrition, physical activity, and healthy GWG support less or not at all when women presented with other issues such as substance use. In contrast, GWG monitoring and weight-related behaviour support were more likely to be offered to women who presented with overweight or obesity. Clinicians who provided the continuity of care reported engaging in these practices more often compared to those who worked with non-continuity-of-care models. A lack of relevant weight-related, nutrition, and behaviour change training for clinicians was common, and no viable referral pathways for dietetic support was available to provide additional expert nutrition support to women within the public health system. We found that clinicians wanted access to patient information that would better engage women with low literacy and health literacy levels, women experiencing socioeconomic disadvantage, and women from different cultural and linguistic backgrounds.

Our findings that nutrition and physical activity support was given lower priority in the face of competing issues, or if a woman’s weight was viewed to be within a normal weight range, are consistent with other studies [44,45]. However, these gaps in health behaviour support can reinforce poor chronic disease outcomes for women and their babies [46,47]. Gaining weight in pregnancy above or below recommendations is associated with increased adverse pregnancy and health outcomes for mothers and babies, even if a woman begins her pregnancy without overweight or obesity [48]. Monitoring GWG and providing the related health behaviour advice to women based on their weight status may be indicative of a weight stigma and inadequate knowledge about the adverse effects of GWG outside of recommendations, regardless of pre-pregnancy weight [45,49]. These factors also mediate how clinicians prioritise GWG conversations when there are numerous other issues to address. However, variation in weight support provision may be more pronounced for women living with social and economic disadvantage who experience an increased burden of antenatal risk factors, including overweight and obesity, excess or inadequate pregnancy weight gain, and undernutrition [36,50,51,52,53,54]. These women can face challenges to maintain a healthy diet and physical activity practices due to financial constraints, lack of knowledge and skills, and welfare concerns including family violence, which can add to the complexity and time needed to advise and assist women in antenatal appointments [55,56]. While a lack of time in appointments is cited as a common barrier to GWG counselling [19,26], beliefs by clinicians that their ability to influence GWG-related behaviours is low and that women are more influenced by their family, habits, and background can also impact on whether support is offered [44].

These results highlight important opportunities for workforce capacity building and health system changes for weight- and nutrition-related support. The reported lack of training in behaviour change techniques and content such as nutrition and physical activity in pregnancy is consistent with other literature [14,57,58]. Professional development training can improve clinician awareness of GWG health consequences and the management of healthy weight gain [59], the importance of appreciating the effects of weight stigma [27], and the ability and confidence to support behaviour change through brief behaviour change interventions during antenatal appointments [60,61]. Our study emphasised the need to supplement this support with timely access to dietitians to whom clinicians could make referrals, particularly for the women they see with complex issues. Nevertheless, access to maternal dietetic services during pregnancy is often limited across Australian publicly funded settings [62], despite evidence of improved health outcomes for women, including those with a lower socioeconomic status who might be unable to afford or access these services outside of the public health system [63,64,65]. Increasing accessibility of specialised nutrition advice for women living with social and economic disadvantage has been shown to help identify the barriers and solutions for health eating relevant to their circumstances [66,67]. Moreover, the preference by clinicians for dietetic support to be part of on-site multidisciplinary teams to assist with catching women ‘in the moment’ would help overcome barriers women might have to accessing health services such as financial, time, or transport constraints and a lack of social support [68]. Finally, improving access to appropriate information and resources for women that are sensitive to social inequalities [63,66,69], health literacy levels [30], and culturally appropriate for migrant women [67,70,71] was seen as important, to recognise the social and environmental influences on a woman’s behaviour and motivations when supporting behaviour change.

The results from our study showed that GWG monitoring and weight-related counselling was more difficult and less consistent where there was no continuity of care, an acknowledged health systems barrier to health behaviour change support [25,72]. Across the service, the opportunity to be weighed was not offered to all women at every appointment, but clinicians providing continuity of care would use visual cues to prompt a weighing being offered or a weight-related discussion. Consistent with other studies, these clinicians were also more comfortable monitoring weight and offering individualised support throughout pregnancy because of the ongoing therapeutic relationship with women [72]. However, clinicians with these models were more likely to be seeing women with lower-risk profiles, and less likely to have to prioritise GWG or nutrition support around more complex issues. These factors all contribute to inconsistencies in weight-related practices and support across the different models of care.

At the time this research was conducted, the complex care and high risk clinics at the hospital were not structured to provide women with the continuity of care, compounding the difficulties prioritising and providing weight gain and nutrition support. Most antenatal clinicians working in the Australian public system are similarly navigating antenatal care within fragmented care models. Nationally, almost 60% of models of care in public hospital maternity care have no continuity of care [73]. Clinicians in our study working in fragmented care made attempts to see the same women across appointments to create some continuity, in part to make appointments easier for women with complex issues and to support better health and wellbeing outcomes. The benefits of continuity of care are well-recognised for women, their babies, and clinicians, [74,75,76], and have been shown to be important for women with a low socioeconomic status and social risk factors [77]. Thus, our results support the ongoing advocacy for health system changes to increase access to the continuity of care to help overcome health inequalities [74,77,78,79].

The study findings should be considered in the context of some strengths and limitations. The strengths include the inclusion of midwives and obstetricians with a wide range of professional experience who worked across all models of care, including low and high risk clinics. The interviews were conducted by an experienced researcher, using an interview schedule adapted to suit our local context in consultation with antenatal clinicians on our investigator team. The setting was the only tertiary centre for maternal health care in the state of Tasmania and the findings may be generalisable to similar settings. The number of clinicians interviewed reflects a small service relative to other settings, and the formative work undertaken in this project serves as a pragmatic case study of using research and evidence to inform health system change. The limitations include the use of convenience sampling, where clinicians volunteered to be interviewed. This means that interview participants were potentially more interested, confident, and engaged in weight-related behaviour change support compared to clinicians who did not volunteer. This may have resulted in the underreporting of some experiences and barriers. All interviewees were female, which was representative of the predominantly female workforce, but the study does not represent the experiences and views of male clinicians which might differ with respect to weight-related beliefs and practices. Some studies suggest potential variations in communication styles and decision-making approaches between male and female healthcare providers. For example, a study of primary healthcare providers in the United States showed that female providers were more likely to provide nutrition advice and counselling related to weight control [80]. The barriers related gender differences in how maternity care is provided is an emerging field and requires further investigation.

## 5. Summary of the Key Findings and Recommendations

These recommendations aim to create an antenatal care environment that is supportive, unbiased, and well-equipped to address weight management and nutrition for all pregnant women, regardless of their background.

Key Findings:
  - Healthy gestational weight gain (GWG) and nutrition discussions are often overshadowed by other health concerns;
  - Care and advice regarding healthy GWG and nutrition can vary depending on a woman’s weight;
  - Continuity of care facilitates open and ongoing conversations about healthy GWG and nutrition;
  - Increased resources such as access to dietitian referrals are needed for weight and nutrition support in the public health system;
  - Tailored education resources are critical for women with diverse social and economic backgrounds and literacy levels.
Recommendations:
  1. Embedding in routine care:
    - Integrate healthy GWG and nutrition support into routine antenatal care, addressing it alongside other health concerns;
    - Frame healthy weight gain and good nutrition as positive factors for both the mother’s and baby’s well-being.
  2. Standardised care:
    - Develop evidence-based weight and nutrition support protocols for all pregnant women, regardless of their weight;
    - Train antenatal clinicians on these protocols to ensure consistent, unbiased, and non-stigmatising care.
  3. Continuity of care:
    - Advocate for system changes to increase access to continuity-of-care pathways that allow for ongoing conversations about weight, nutrition, and overall health.
  4. Build capacity:
    - Provide training to increase the ability and confidence of clinicians to support health behaviour change through brief behaviour change interventions;
    - Increase access to dietetic support for pregnant women in publicly funded health care.
  5. Tailored support:
    - Create resources in various formats (written, audio, visual) catering to different literacy and health literacy levels;
    - Translate materials into common languages spoken within the local population;
    - Consider cultural, social, and economic sensitivities when developing resources and providing support.

## 6. Conclusions

Our research emphasises the challenges faced by antenatal clinicians working in public maternity health systems. There is an imperative to better understand the factors that impede healthy nutrition, physical activity, and weight gain support in publicly funded antenatal care, particularly in health systems where clinicians are more likely to be supporting women with higher levels of socioeconomic and other burdens. Alongside the need for improved access to pre-clinical and post-qualification behaviour change training, clinicians were often overstretched when balancing the health, clinical, and psychosocial needs of women, and would benefit from an integrated multidisciplinary team input from disciplines such as dietetics. Our findings can be used to advocate for health system changes to improve access to referral pathways and increase access to continuity-of-care models for women with higher levels of complex needs or health issues. Ready access to patient resources that are appropriately tailored to the diverse socioeconomic, cultural, and literacy needs of women would also better enable clinicians to effectively support diet and physical activity behaviour changes in antenatal care.

## Data Availability

Data are not available due to ethical restrictions.

## Supplementary Materials

The following supporting information can be downloaded at: https://www.mdpi.com/article/10.3390/nu16091251/s1, Table S1: Interview schedule.
