# Supplementary material for: Health System and Individual Barriers to Supporting Healthy Gestational Weight Gain and Nutrition: A Qualitative Study of the Experiences of Midwives and Obstetricians in Publicly Funded Antenatal Care in Tasmania, Australia
Source: Nutrients. 2024 Apr 23;16(9):1251. doi: 10.3390/nu16091251 (PMC11085055; doi:10.3390/nu16091251)
Supplement: Supplementary file 1 [file nutrients-16-01251-s001.zip › nutrients-2941161-supplementary.pdf]

Supplementary Table S1

Introduction	**Prompts**
1. Tell me a bit about what you do here in the service?	-How many years of experience do you have in this field?<br>-How long have you worked in this service?<br>-Types of women (low risk, complex cases etc)<br>-Different models of antenatal care
THEME 1 Routine care (across all models of care)	
2. When do you usually see a woman for the first time?	-Stages of pregnancy (booking in, delivery)<br>-On average, how many times might you see the same woman throughout her pregnancy? (Continuity of care)
3. What usually happens when you see a woman for the first time in her pregnancy?	-What does the assessment/visit involve?<br>-What do you talk about?<br>-How do you think that information is usually received?<br>-What do the women usually want to talk about?<br>-Do you weigh the woman?<br>-What health-related behaviours do you discuss? Mental health?
4. Do you specifically discuss nutrition and exercise in pregnancy with them?	-How is this usually received by the woman?<br>-What advice do you give?
5. Do you specifically discuss weight, or healthy weight gain in pregnancy?	-If no, why?<br>-If yes, how is this usually received by the woman?<br>-What advice do you usually give?<br>• Appropriate weight gain<br>• Different advice for women of different pre-pregnancy weights?<br>-How do you judge healthy pregnancy weight gain?<br>• Guidelines<br>• measuring weight gain<br>• Other methods, such as visual?
6. How are these issues followed up during pregnancy?	-If a woman is gaining outside weight gain guidelines (too much or too little), what would you do?<br>Ability to refer to dietitian or allied health professional?
7. Would you know if these topics have been raised in previous visits with	Continuity of care and advice<br>• Known, because working in continuity of care, or ask

other clinicians, and what has been discussed?	• Records – Obstetrix or patient notes
THEME 2 Weight-related behaviour change	
8. How do you feel talking to women about nutrition and exercise in pregnancy?	-Do you feel confident to give advice or support women to make changes?<br>-What would make the conversation easier?<br>-How does supporting weight-related behaviour change (WRBC) apply to women with more complex life circumstances or needs?
9. How do you feel talking to women about weight, and weight gain in pregnancy?	-Do you feel confident to give advice or support women to make changes?<br>-What would make the conversation easier?<br>-How does supporting weight-related behaviour change apply to women with more complex life circumstances or needs?
10. How does the availability of resources/training influence your support of women?	-Time available for appointments<br>-Referral options<br>-Access to equipment (scales etc) and resources in community-based antenatal clinics vs centrally located clinic near the hospital<br>-Patient resources at appropriate health literacy levels<br>-Staff training
11. Can you tell me about some times where women have made positive WRBC during pregnancy?	What do you think helped?<br>-Motivations, perceptions, supports available, economic and social advantage or disadvantage
12. And those who haven't made positive WRBC, what do you believe were the barriers?	-Motivations, perceptions, supports available, economic and social advantage or disadvantage
13. What do you think would help women to change their weight-related behaviours?	-Behaviour change support in antenatal care<br>-Technology-based behaviour change support - Online information or forums, mobile phone apps etc<br>-Links to community supports and government programs
14. Any other comments or suggestions on how behaviour change could be supported during pregnancy?	

Interview schedule adapted (with permission from the authors) from Flannery C, McHugh S, Kenny LC, O'Riordan MN, McAuliffe FM, Bradley C, et al. Exploring obstetricians', midwives' and general practitioners' approach to weight management in pregnant women with a BMI ≥25 kg/m2: a qualitative study. BMJ Open. 2019;9(1).
